# Morphological variation and reproductive isolation in the *Hetaerina americana* species complex

**DOI:** 10.1038/s41598-022-14866-8

**Published:** 2022-06-28

**Authors:** Yesenia Margarita Vega-Sánchez, Luis Mendoza-Cuenca, Antonio González-Rodríguez

**Affiliations:** 1 Instituto de Investigaciones en Ecosistemas y Sustentabilidad, Universidad Nacional Autónoma de México, 58190 Morelia, Mexico; 2 Facultad de Biología, Universidad Michoacana de San Nicolás de Hidalgo, 58030 Morelia, Mexico

**Keywords:** Ecology, Behavioural ecology, Evolutionary ecology

## Abstract

Incomplete premating barriers in closely related species may result in reproductive interference. This process has different fitness consequences and can lead to three scenarios: niche segregation, sexual exclusion, or reproductive character displacement. In morphologically cryptic species, isolation barriers can be difficult to recognize. Here, we analyzed the morphological, behavioral, and genetic differences between two sympatric cryptic species of the genus *Hetaerina* to determine the characters that contribute the most to reproductive isolation and the effect of the high rates of behavior interference between the species. We found complete genetic isolation and significant differences in the morphometry of caudal appendages and wing shape, as well as body size variation between species. In contrast, we did not find clear differences in the coloration of the wing spot and observed high rates of interspecific aggression. Our results suggest that divergence in the shape of the caudal appendages is the principal pre-mating barrier that prevents interspecific mating. Moreover, a scenario of character displacement on body size was found. Nevertheless, size could play an important role in both inter- and intrasexual interactions and, therefore, we cannot differentiate if it has resulted from reproductive or aggressive interference.

## Introduction

In sympatric closely related species, there may be costs associated with incomplete premating barriers^1^. When there is incomplete species or mate recognition, processes of reproductive interference can occur. Reproductive interference is any interspecific intersexual interaction with a negative effect on the fitness of the species involved^2,3^, resulting from wasted time, energy, nutrients or gametes. These fitness consequences depend on the type of reproductive interference: signal jamming, misdirected courtship, heterospecific mating attempts, erroneous female choice, heterospecific mating and hybridization^2^. Moreover, similar to competition, reproductive interference is density-dependent and can lead to three principal scenarios: niche segregation, sexual exclusion or reproductive character displacement^2–5^. Niche segregation refers to temporal or spatial habitat partitioning to avoid interspecific interactions^2,6^, while sexual exclusion is the demographic displacement of one of the involved species (local extinction)^7^. Finally, reproductive character displacement is a divergence in traits related to the species recognition systems in sympatric conditions^2,8^. The last two processes may be a consequence of reproductive interference in a longer evolutionary lapse^7^.

*Hetaerina* is a new world genus where all male individuals present a red wing spot on the base of each wing and display territorial behavior, characteristics that are absent in females^9^. Since *Hetaerina* females do not select males, and territorial males (i.e., sexually mature adults) do not respond aggressively to immature males, this strongly suggests that the evolution of wing pigmentation is driven by recognition between male competitors^10–14^.

Moreover, reproductive interference (i.e., heterospecific mating attempts) has been documented to occur at high rates between some *Hetaerina* species (e.g., *H. americana*, *H. occisa, H. cruentata*) due to both a high frequency of species in sympatry and the similarity of the wing coloration of the females^11–13^. Also, aggressive interference is common in *Hetaerina* males^13,15^. Unlike reproductive interference, aggressive interference includes interspecific aggressive interactions such as fights, displays and territoriality, and can drive different evolutionary scenarios such as agonistic character displacement or competitive exclusion^3^. For *Hetaerina*, when aggressive interference occurs between species with contrasting wing pigmentation as with *H. titia* and *H. americana*, a process of agonistic character displacement has been suggested as a way to avoid these interspecific interactions^13^. However, interspecific competition among *Hetaerina* males mostly involves species with similar wing spot coloration (e.g*., H. americana, H. occisa, H. cruentata*)^10^.

*Hetaerina americana* had been recognized as a widely distributed species until very recently, when a cryptic species complex was suggested for the American rubyspot, possibly integrated by three species^16^. Moreover, this “species” has been extensively used as an ecological model for behavioral studies and diverse mating tactics have been described. In this paper, we studied two cryptic species of the American rubyspot complex that are formally described: *H. americana* and *H. calverti*. *Hetaerina americana* presents an extensive distribution from Chiapas in Mexico to Canada; in contrast, *H. calverti* is distributed in Honduras, El Salvador and Guatemala to the north of Mexico. The two species frequently occur in sympatry^16,17^. Furthermore, these species have indistinguishable coloration patterns to the naked eye and the diagnostic character for males is the shape of the superior caudal appendages; for females, morphological differentiation is less clear, and a genetic assignment is needed^17^. Therefore, these species are an excellent model to assess the effects of reproductive interference and make some inferences about the mechanisms that could maintain reproductive isolation through the detailed evaluation of differentiation in morphological characters such as wing coloration and the shape and size of caudal appendages, which are characters that contribute to species recognition in other odonate species^18–20^.

Our main questions were (1) Is there complete reproductive isolation between the two cryptic species when they occur in sympatry? (2) If this is the case, how is this reproductive isolation maintained? (3) Which morphological characters are important for reproductive isolation? And (4) what is the evolutionary effect of interference between these two species? To assess these questions, we explored whether there is behavioral and morphological variation between *H. americana* and *H. calverti* and tested the possibility of hybridization with genetic markers, all under sympatric conditions.

## Results

### Genotyping and genetic diversity

Microsatellite data were obtained from 162 individuals. However, the H17 locus had to be discarded because it did not amplify in most of the individuals of *H. calverti*. In total 14 tandems were collected: 11 for the year 2017, and three for the year 2018. Of these, eleven corresponded to male individuals of *H. calverti* and three to male individuals of *H. americana.* The genotyping corroborated that none of these tandems corresponded to heterospecific mating.

The genetic diversity estimators showed similar values between species (Table 1). The inbreeding coefficient (*F*) was very high and significant in *H. calverti* and nonsignificant in *H. americana* (Table 1). According to FreeNA, there was evidence of null alleles at three loci of *H. calverti*, which could explain the high *F* values. Genetic differentiation was very high between the two species (F_ST_ = 0.72; *p* < 0.0001) without an important effect of null alleles on the estimation (corrected F_ST_ = 0.76).

**Table 1:** Summary of genetic diversity estimators for *H. calverti* and *H. americana* individuals in sympatry at Apazapan, Veracruz.

Species	N	Na (SE)	Ne (SE)	Ho (SE)	He (SE)	uHe (SE)	F
*H. calverti*	100	4 (0.89)	1.29 (0.01)	0.11 (0.05)	0.21 (0.06)	0.21 (0.07)	0.836*
*H. americana*	57	2 (0.55)	1.36 (0.22)	0.12 (0.11)	0.19 (0.11)	0.19 (0.11)	0.035

N = sample size; Na = mean number of different alleles; Ne = mean number of effective alleles; Ho = mean observed heterozygosity; He = mean expected heterozygosity; uHe = mean unbiased expected heterozygosity; F = mean fixation index; SE = standard error.

*p values < 0.0001 after 10,000 permutations.

**Figure 1:**
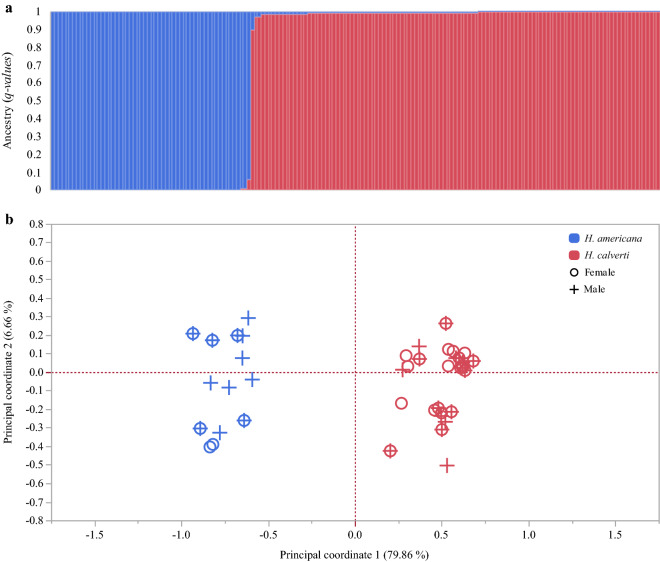
Genetic structure analyses. (**a**) STRUCTURE assignment analysis for K = 2 from five nuclear microsatellites for sympatric *H. calverti* and *H. americana* individuals in Apazapan, Veracruz. In the bar plot, individuals are represented by thin vertical lines, which are partitioned into K shaded segments representing each individual’s estimated membership fraction. (**b**) Principal coordinate analysis (PCoA) for females and males collected in both years.

For the genetic assignment, only 10 collected females corresponded to *H. americana* and 27 to *H. calverti* for the year 2017 and seven to *H. americana* and 33 to *H. calverti* for the year 2018. Three females were not identified due to problems with DNA quality. Individual *q-values* showed that mixed ancestry is almost nonexistent (only one individual showed 90% of one genetic group and 10% of the other). Therefore, if hybrid individuals are present, they should be at a very low frequency. The PCoA analysis obtained based on the genetic distances among all individuals also showed that there are two highly differentiated groups, which agree with the genetic groups suggested by STRUCTURE (Fig. 1).

### Abundance and behavioral interactions

For the year 2017, 220 individuals were marked, and differences in the abundance of the two cryptic species were observed. In total, 60 males of *H. americana* and 160 males of *H. calverti* were marked. In the year 2018, a lower abundance of both species was found: 146 individuals were marked in total, although the proportions were more equitable: 54 males of *H. americana* and 92 of *H. calverti*.

We found interspecific differences in the number of days that males maintain a territory but only in one of the years. The following results are presented as mean ± SE. Males of *H. americana* maintained their territories for more days, during the year 2017 but not in the year 2018 (Supplementary Fig. S1; year 2017*: H. americana* 4.09 ± 0.53, *H. calverti* 1.44 ± 0.29, X^2^ = 22.11, *p* < 0.0001; year 2018: *H. americana* 2.14 ± 0.14, *H. calverti* 2.36 ± 0.43, X^2^ = 0.07, *p* = 0.80). We also found differences between both species in the average time duration of aggressive interactions depending on the opponent species and the year. In the year 2017, for the territorial males of *H. calverti*, aggressive intraspecific interactions were longer than interspecific interactions with males of *H. americana* or *H. occisa* (species also present at this site) (X^2^ = 8.33, *p* = 0.017; Supplementary Fig. S1; Supplementary Table S1). In the case of territorial males of *H. americana*, aggressive interactions were longer with males of *H. calverti* than with males of their species or males of *H. occisa* (X^2^ = 7.20, *p* = 0.027; Supplementary Fig. S1; Supplementary Table S1). In the year 2018, no significant differences were found in the duration of aggressive territorial interactions in either *H. calverti* or *H. americana* (Supplementary Fig. S1; Supplementary Table S1).

### Variation in secondary sexual traits

#### Differences in size between species

**Figure 2:**
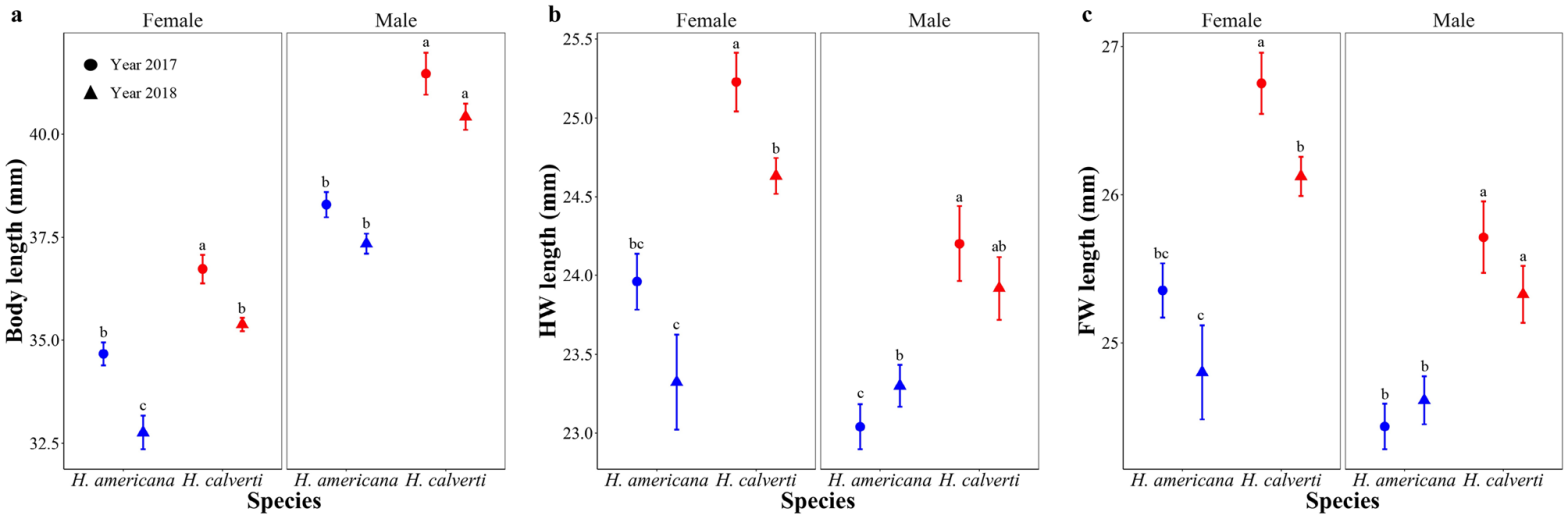
Variation in size between species. Differences in body length (**a**), hind wing length (**b**) and fore wing length (**c**) between species and years of sampling by sex. Means and standard errors are shown, different letters represent significant differences.

The two species differed significantly in size (Fig. 2; Supplementary Table S2 and S3). Males and females of *H. calverti* had larger wings and greater body length than males and females of *H. americana* regardless of the year of sampling (Fig. 2). Moreover, we found that there is an effect of the year of sampling on the female body length since individuals collected in the year 2017 were larger than those collected in the year 2018 (Supplementary Tables S2 and S3), but differences between species remained.

Additionally, the linear mixed effect model for comparing the body length of both species between localities under conditions of sympatry or allopatry showed significant differences (Species: Estimate = 0.79, E.E. = 0.34, t-value = 1.14, *p* < 0.0001; Locality type: Estimate = 0.44, E.E. = 0.56, t-value = − 0.78, *p* = 0.89; Interaction Species*Locality type: Estimate = 1.51, E.E. = 0.72, t-value = 2.07, *p* = 0.037). We found that differences in body length are enhanced in sympatry, with males of *H. calverti* being larger (41.04 ± 0.17 mm) than males of *H. americana* (38.90 ± 0.13 mm) (Supplementary Fig. S2).

#### Wing morphology and coloration patterns of wing spots

The Procrustes ANOVA conducted to determine shape variation both for hindwing (HW) and forewing (FW) in males showed significant differences between years of sampling and species but not for the interaction (Supplementary Table S3). Post-hoc multiple comparisons showed significant differences between species both in the same year of sampling and between years. The Procrustes ANOVA analysis used to determine interspecific shape variation in females was also significant for the year of sampling and species for both HW and FW (Supplementary Table S4). Post-hoc multiple comparisons only showed significant intra- and inter-year differences between species. The principal component analyses showed that the species present different wing shapes for both sexes (Supplementary Fig. S3).

Regression results showed no allometric effect on wing shape for males (*H. calverti* HW, R^2^ = 0.013, *p* = 0.713; *H. calverti* FW, R^2^ = 0.042, *p* = 0.06; *H. americana* HW, R^2^ = 0.043, *p* = 0.104; *H. americana* FW, R^2^ = 0.045, *p* = 0.086) nor females (*H. calverti* HW, R^2^ = 0.013, *p* = 0.713; *H. calverti* FW, R^2^ = 0.044, *p* = 0.172; *H. americana* HW, R^2^ = 0.024, *p* = 0.171; *H. americana* FW, R^2^ = 0.063, *p* = 0.06).

**Figure 3:**
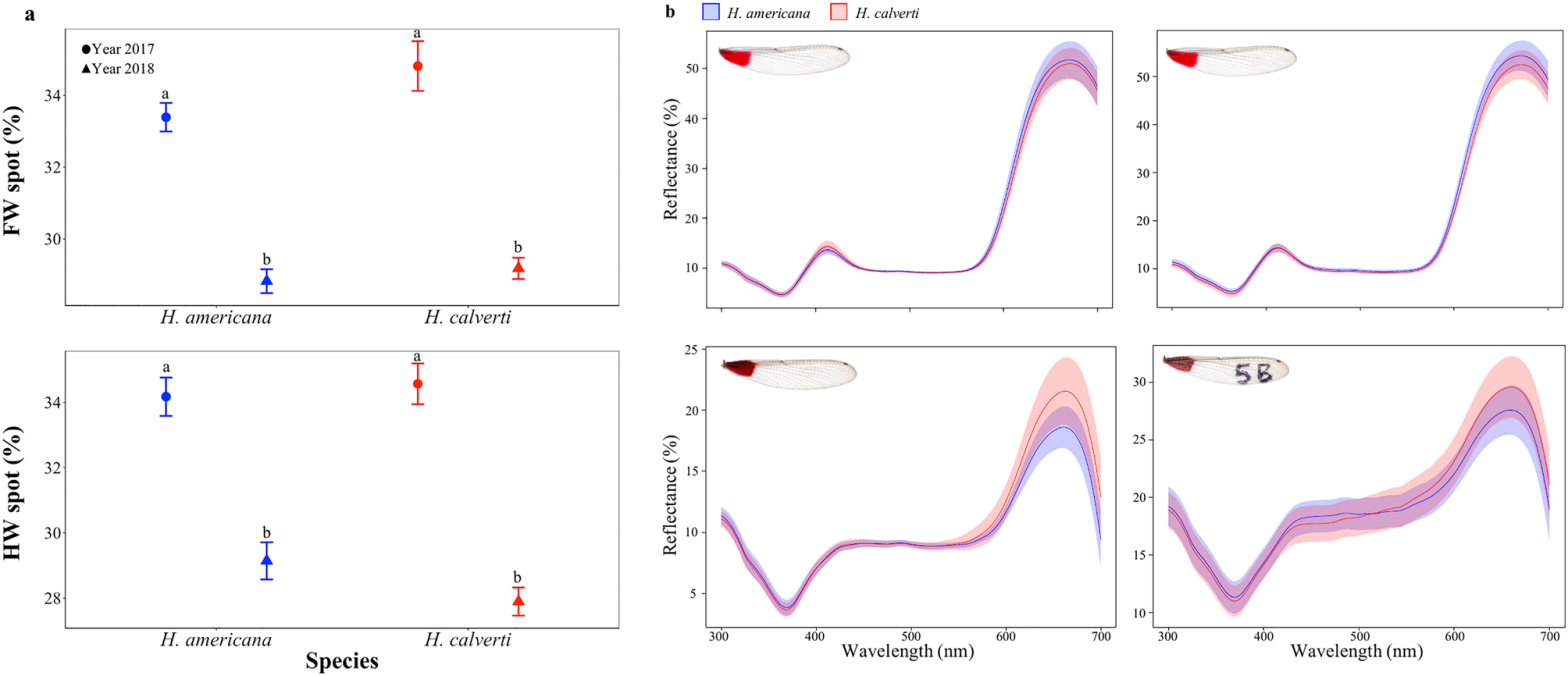
Variation in wing spots between species. (**a**) Variation in the percentage of FW (above) and HW (bottom) spots between species and years. (**b**) Reflectance patterns of FW (above) and HW (bottom), including inner (left) and outer (right) parts.

Related to coloration, we found no differences in the percentage of the wing spot with respect to the total wing area between species (Fig. 3a), but we observed a great difference in the percentage of the wing spot related to the year of sampling, with larger spots in the year 2017 (Supplementary Table S3; Fig. 3a). The comparison of reflectance spectra between the species showed an almost complete overlap for the FW, while, for the HW, it is possible to observe a subtle variation, especially in the peak of the long wave (670 nm) spectral region (Fig. 3b). Concordantly, our color analysis predicted that the vision system of *Calopteryx splendens* is not capable of discriminating chromatic differences between *H. calverti* and *H. americana* males. The JND values comparing both species were not above the discrimination threshold (i.e., JND > 1) for the *Calopteryx* vision model for either FW (JND_inner_ = 0.262, JND_outer_ = 0.123) and HW (JND_inner_ = 0.432, JND_outer_ = 0.433).

#### Variation in superior caudal appendages

**Figure 4:**
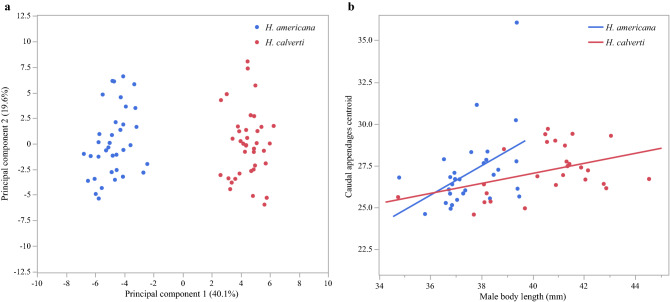
Differences in shape and size of caudal appendages. (**a**) Principal components analysis for the shape of superior caudal appendages of 77 males. (**b**) Relationship between the body length and the size of caudal appendages for each species.

The two first principal components of the PCA analysis using the Procrustes coordinates that describe the shape of the superior caudal appendages recovered 59.7% of the variation and showed two distinct groups, representing each species (Fig. 4a). These groups are also different in the discriminant analysis (Wilks’ Lambda: F = 73.82, *p* < 0.0001).

We also found a positive relationship between male body length and the size of caudal appendages (Fig. 4b) (*H. americana*: R^2^ = 0.21, F = 7, *p* = 0.0134; *H. calverti*: R^2^ = 0.17, F = 5.19, *p* = 0.0315).

## Discussion

The mate/species recognition systems in Odonata are complex, and usually several sexual characters are involved. These characters could act as barriers (that are mostly pre-zygotic) hence affecting the reproductive isolation process. These characters could be body and wing coloration (i.e., behavioral/sexual isolation, e.g., *Calopteryx*), the shape of the male caudal appendages and the female prothorax (i.e., mechanical/sensorial isolation, e.g., *Enallagma*, *Ischnura*) and even the shape of the secondary male genitalia (i.e., mechanical/sensorial isolation, e.g., *Calopteryx*, *Polythore*, *Chalcopteryx*, etc.)^19–21^. Besides these characters, different behaviors such as territoriality and complex courtships can be involved in the mating process and species recognition systems^22^.

In this study, the high genetic differentiation and the results of the Bayesian genetic assignment suggest that there is no hybridization between the two cryptic species and, therefore, that reproductive barriers are effective. It has been proposed that in sympatric species with overlapping mating seasons and that interact with each other (e.g., competition for territories), but do not hybridize, the existence of behavioral/sexual reproductive isolation could be inferred^1^. However, our results indicate that reproductive isolation between *H. calverti* and *H. americana* is not related to wing coloration which supports the idea that the wing spots have evolved through male–male competition.

Then, how is the reproductive isolation between these species maintained? The process of choosing a mate in *Hetaerina* involves two main phases: (1) the male’s choice, which begins when a female flies into a male’s territory and ends when a male grasps the female in flight without prior courtship. There is a high degree of reproductive interference (heterospecific mating attempts) between *Hetaerina* species, especially when the females have similar wing colorations (e. g. females of *H. occisa* and *H. americana*), but lessened interference when females are contrasting in wing coloration (i.e. females of *H. titia*, smoked-wing morphotype)^23^, suggesting that males cannot discriminate similar females. Although in our case the females of *H. calverti* and *H. americana* are very similar in color patterns, we cannot discard that males may be able to differentiate heterospecific females. This is partially supported by the absence of heterospecific tandems in our observations, which suggests that another character could be involved in the recognition process when the males try to grasp a female in flight. However, the number of tandems analyzed is low and, thus, more data are necessary to make more robust inferences about possible male mate recognition prior to clasping. (2) The female’s choice, which starts when the male has already chosen a female and the couple is in the tandem position. For other calopterygid species, wing coloration is the main character by which females recognize males^24^, but this does not occur in *Hetaerina*^13^. The slight variation in wing spot coloration between these cryptic species supports this notion. Another character involved in species recognition and mate choice in odonates is the shape of the caudal appendages, and the females decide whether to copulate depending on the mechanical stimulation of these structures^18,22,25^, and it is precisely this character that presents the greatest divergence between these cryptic species. Mechanical/sensorial isolation barriers contribute most to reproductive isolation in some odonates, especially in species that do not exhibit courtship or territorial behavior, such as many coenagrionids, although this barrier rarely leads to complete isolation by itself^19^. Other pre- and postzygotic barriers such as gametic and genetic incompatibility could also contribute to the complete reproductive isolation, which can be expected in highly diverged species like in this case^17^. Nevertheless, to evaluate these aspects, experimental analyses with different crosses are needed.

Even though behavioral/sexual isolation based on male wing coloration has been ruled out for *Hetaerina*^10,13^, our results suggest that characters other than color must be involved in the reproductive isolation of these cryptic species. For example, body size was previously reported as a strong premating barrier in different animal groups^26–28^. In this context, male assortative mating by size has been described in *H. americana sensu lato*^29^, and even though we cannot ensure interspecific discrimination of females based on their size, it has been widely recognized that assortative mating can promote or reinforce divergence even in incipient species and in the presence of gene flow^30,31^. Assortative mating in males of *H. americana* y *H. calverti* could have two causes: first, males prefer mates with specific trait values, in this case larger females because they may be more fertile (and not because they discriminate between species). Second, mating with females that match their own phenotype, for example, large or small males may be able to mate only with females of a similar size due to the isometric relationship between body size and the size of the caudal appendages, since, although the male may be capable of forming the tandem with the female and the shape of the caudal appendages may be adequate, the variation in size between these structures could modify the stimulation in the females and result in an unsuccessful mating attempt^25^. Moreover, males cannot force females to copulate^32^. Also, during the whole mating process, males should avoid harassment from other males (they attempt to break up the tandem and steal the female) and take the female to oviposition sites. This may be more likely if there is not a big difference in the body size between individuals.

Moreover, size has an important effect on competition between males^33^. Larger males can maintain a territory longer and therefore have more mating opportunities^34,35^. However, all studies to date have considered *H. americana* as a single species^29,35–37^ and it is unknown which is the effect of the possible presence of cryptic species at the localities where the studies were carried on. We suggest that body size may be an important character that usually is not taken into account in analyses of interspecific interactions, but experimental studies would be needed to define its role in both inter- and intrasexual interactions.

Wing shape and body size have a critical aerodynamic effect on flight performance^38–40^. The variation in flight performance has been related to behaviors such as territoriality and courtships, and despite the fact that in *Hetaerina* there is no courtship, this wing shape variation could be associated with the recognition of conspecifics in flight (e.g., before forming a tandem).

Behavioral interference (i.e., reproductive and aggressive interference^3^) can lead to several patterns, such as species segregation into particular habitats, reproductive or agonistic character displacement, and sexual or competitive exclusion^3^. Drury et al.^11^ suggested the catch-22 hypothesis, which states that if the females of sympatric species are similar phenotypically (in terms of coloration), males will be unable to discriminate between them. Therefore, the interference will remain indefinitely since there is no selection on trait divergence related to the recognition of mates by males. However, our results indicate phenotypic variation and even a character displacement pattern associated with body size.

The character displacement pattern on body size could help to reduce interference but in the case of heterospecific rivalry interactions, results suggest that differences in body size do not necessarily reduce the intensity of aggressive interspecific interactions. Then, the variation on body size may be related to reduce reproductive interference, but this needs to be tested.

Other effects of behavior interference such as niche segregation or sexual or competitive exclusion may have evolved in these species. Niche segregation has been reported for *H. cruentata* and *H. occisa* in sympatry with *H. americana* because the former prefers shadier territories than the latter, avoiding the interspecific encounters^6^.

Finally, a latent exclusion (the demographic displacement of one of the involved species; local extinction) could be taking place because *Hetaerina americana* has the lowest abundance at the site, even lower than *H. occisa*, which could be as common as *H. calverti* (Y. M. Vega-Sánchez, personal observation). In addition, *Hetaerina americana* males are smaller, and males of *H. calverti* could displace them because body size is an important character that predicts males’ success in maintaining a territory and therefore of their fitness. Interestingly, we observed that males of *H. americana* remained in territories for a longer time (although only in year 2017). However, it has been suggested that this may happen when the territories vary in quality^41^, for example, males established in shadier territories that have fewer passing females should experiment less aggressive interactions than males established in better territories and then remain longer in their territories^6,33^. We need to analyze more sympatric localities to test if this process, a possible competitive exclusion, is occurring.

In conclusion, we found, for the cryptic species studied here, that caudal appendages seem to be the principal trait related to the avoidance of heterospecific mating. However, understanding why other traits such as coloration (as in other *Hetaerina* species) have not diverged between cryptic species remains an unanswered question. Moreover, we showed that body size also may be an important character that could enhance reproductive isolation, reducing heterospecific mating. Besides, body size could play an important role in intrasexual aggressive interactions. Nevertheless, more data are needed to analyze the evolutionary and ecological importance of size in these organisms.

## Materials and methods

### Sampling

The study was conducted at Apazapan, Veracruz, Mexico (19°19′30.14″ N, 96°43′29.63″ W), in a perennial tributary stream of the Pescados River. Using mark-recapture techniques, we studied both species in two field campaigns, the first in April–May 2017 (hereafter year 2017) and the second in February 2018 (hereafter year 2018), on average 12 days per year. We marked all male individuals and assigned them to one of four age categories: teneral, young, mature and old; these categories are determined by the appearance of the wings (stiffness, brightness, damage)^42^. Moreover, we captured individuals that were found in tandem and that had copulated. At the end of each campaign, we collected 20 male individuals of each species and 40 females (females cannot be separated into species due to the lack of diagnostic characters). We used all these individuals to analyze genetic and morphological variation (see below).

### Genetic diversity and gene flow

We genotyped all males and females (including tandems) collected at the end of each campaign to determine if there is evidence of genetic exchange between the two cryptic species. The genetic analysis was performed using the same six microsatellite loci and protocol used by^16^. With this data, we obtained different genetic diversity estimators: number of alleles, number of effective alleles, observed and expected heterozygosity, unbiased expected heterozygosity and the interbreeding coefficient using GenAlex v. 6.0^43^. We also estimated the null allele frequencies for each locus and species in FreeNA v.1^44^.

To estimate the degree of genetic differentiation among the two species we performed an analysis of molecular variance (AMOVA) in Arlequin v.3.5^45^. Additionally, Bayesian analysis in STRUCTURE^46^ was performed, which allowed to determine the genetic ancestry (*q-value*) for each individual and evaluate the possibility of genetic admixture, which would support the occurrence of hybridization between the two species. The genetic relationships between individuals were also visualized by a Principal Coordinates Analysis (PCoA) using the GenAlEx program.

### Behavioral interactions between males

We located the territories of all males of both species along one kilometer of the river and marked males on the hindwing with a consecutive number and assigning a letter “A” for *H. calverti* and “B” for *H. americana* (on the basis of the diagnostic characters described by^17^), using a permanent marker. Marked individuals were observed daily, and those males that were present for at least 3 days in the same part of the river were considered territorial^33^. Then, we selected 15 territorial males of each species (i.e., defender individuals) to determine the frequency and duration of their aggressive interactions (intra- and interspecific) during observation periods of 30 min. Non-parametric Kruskal–Wallis, Wilcoxon and Chi-square tests were carried out to assess differences in the duration of the interactions between species and the number of days the males maintain a territory. These analyses were performed for each year separately and carried out in JMP v.15 (SAS Institute Inc).

### Analyses of secondary sexual traits

#### Variation in size

The males and females collected at the end of each campaign were photographed and their four wings were scanned along with a scale. Then, the total body length and the forewings and hindwings length were measured using the Image J software^47^. The total body length was estimated as the straight line from head to the caudal appendages for males and from head to the last abdominal segment for females. Wing length was estimated from the initial part of the costal vein (C) to the end of the radial vein 1 (R1) for both forewing (FW) and hindwing (HW). For these data, two-way analyses of variance (two-way ANOVA) were performed using species and the sampling year as independent variables. The analyses were performed separately for each sex. Post-hoc multiple comparisons were performed using Tukey HSD analyses for each data set.

Moreover, we analyzed the variation in body length for the two species in conditions of sympatry and allopatry through their geographical range using the complete data set of^17^ plus the data obtained in this study since a possible process of reproductive character displacement has been suggested for these species^17^. In total, we measured 602 individuals distributed in 27 allopatric and 12 sympatric localities for *H. americana* and 9 allopatric and 10 sympatric localities for *H. calverti* (Supplementary Table S5). With these data, we performed a linear mixed effects model in the *lme4* package^48^ in the R software, using body length as the response variable, species and locality type (allopatric or sympatric) as fixed effects and the locality as random effect.

#### Variation in wing morphology and coloration

We analyzed wing morphology for all males and females using geometric morphometric techniques. We used the images of individuals that had complete left FW and HW to compare wing shape between males and females of both species in two consecutive years. Ten anatomical marks (i.e., landmarks) were placed on the outline of the FW and HW on the intersection with principal veins (Supplementary Fig. S4), which are homologous landmarks on all individuals of both sexes^40^. Two points were also placed on the millimetric ruler as a scale factor, using Geomorph program version 3.3.2^49^. A Generalized Procrustes Analysis was conducted to obtain the coordinates that were used as variables of shape. We performed Procrustes ANOVA with 9999 permutations, including year and species as independent variables to compare for each sex the shape of FW and HW. We also performed a Principal Components Analysis of the variance–covariance matrix to visualize the wing shape variation separately for each sex.

Multivariate regression was performed to evaluate size effects on wing shape (allometry) using shape as the dependent variable and the natural logarithm of the centroid size as the independent variable for each species, sex, and both wings^50^.

We also compared the percentage area covered by the wing spots with respect to the total wing area, using the ImageJ program for both FW and HW. With these data, we performed two-way ANOVAs in JMP v.15 (SAS Institute Inc) to test for differences between the two species and between years.

The spectral signature of the wing spots of both species was characterized using an Ocean Optics USB2000 spectrophotometer equipped with a xenon pulse lamp (PX2) in a UV–VIS range of 300–750 nm. The spectrometer was calibrated with a diffuse reflectance white standard from Ocean Optics (WS-1). We put the wings on a ColourWorker (X-rite) 80% grey standard chart and obtained these measurements on a 45° angle using an RHP probe support (Ocean Optics, Dunedin, FL). We made four measurements, including the outer and inner part of the right FW spot and the outer and inner part of the right HW spot. These data were obtained for the 28 males collected in the year 2017. We used the package *pavo2* v. 2.6.1 to analyze reflectance spectra^51^. The software allows to manage, process and visualize reflectance spectra in color spaces and psychophysical models for color discrimination, which allows the estimation of potential receptor quantum catch of the wing-spot coloration (i.e., Receptor Noise Limited Model^52^).

In order to evaluate how different both *Hetaerina* species are in terms of their wing spot coloration, we evaluated the ability of an Odonata visual system to discriminate between *H. calverti* and *H. americana* species by their wing spot. Since information about the spectral sensitivities, receptor proportions and receptor noise of *Hetaerina* is not available, we seek the species most closely related to *Hetaerina* for which these data are available. We used pooled sensitivity data of *Calopteryx splendens* (λ_max_ 366, 480, 552 and 640 nm^53^), a receptor proportion of 3:2:2:1 (i.e., ultraviolet, shortwave, mediumwave, and longwave^54^) and receptor noise was adjusted to 0.2 as for other Odonata visual systems^53^. As information for the illuminants, we used the environment "bluesky"^55^ included in the package to model the potential quantum catch of the species. The data for the receptor quantum catch was obtained from fitting a psychophysical visual model of the reflectance spectra of the inner and outer parts of the wings.

We analyzed chromatic contrasts by determining the probability that the *Calopteryx* visual model could discriminate between *H. calverti* and *H. americana* male FW and HW (including inner and outer parts) wing spots. Based on a discrimination threshold of 1 Just Noticeable Difference (JND) tridimensional space (where JND values < 1 imply that the two species are indistinguishable), as commonly used in studies of color discrimination^56^, taking a conservative approach to the interpretation of psychophysical discrimination relative to known discrimination thresholds^57^.

#### The shape of the caudal appendages

To analyze the variation in the shape of the caudal appendages, we took photographs of the superior caudal appendages in a dorsal view of 80 males (20 per species and year) using a stereoscopic microscope with a scale. First a “fan” was superimposed on each image to guide the placement of semilandmarks using MakeFan v.6 program^58^. Then, *x* and *y* coordinates were digitalized at six homologous points of the superior caudal appendage and 22 semilandmarks using TpsDig v.2 program^59^ (Supplementary figure 1 in^16^). These anatomical marks describe the contour shape of the superior caudal appendage in a dorsal view. Once the landmarks and semilandmarks were placed, a Procrustes superimposition was performed using CoorGen v.7 program^58^. Once the coordinates were obtained, they were used as morphological variables to perform principal components and discriminant analyses in JMP v.15 (SAS Institute Inc).

Moreover, we tested for isometric allometry between the male body length and the size of superior caudal appendages using the centroid; we performed a linear regression in JMP v.15 (SAS Institute Inc) for each species.

## Supplementary Information

Supplementary Information.

## Data Availability

The datasets generated and analyzed during the current study are available from the corresponding author upon request.
