# Supplementary material for: Morphological variation and reproductive isolation in the Hetaerina americana species complex
Source: Sci Rep. 2022 Jun 28;12:10888. doi: 10.1038/s41598-022-14866-8 (PMC9240019; doi:10.1038/s41598-022-14866-8)
Supplement: Supplementary file 1 — Supplementary Information. [file 41598_2022_14866_MOESM1_ESM.pdf]

Supplementary information

Morphological variation and reproductive isolation in the *Hetaerina americana* species complex

Yesenia Margarita Vega-Sánchez, Luis Mendoza-Cuenca, and Antonio González-Rodríguez

Supplementary Figure S1. Territorial behavioral variation between species. a) Number of days that males retain a territory. b) Average time duration of aggressive interactions that males of *H. americana* and *H. calverti* spend with conspecific or heterospecific males for the two sampling years. Means and standard errors are show, different letters represent significant differences.

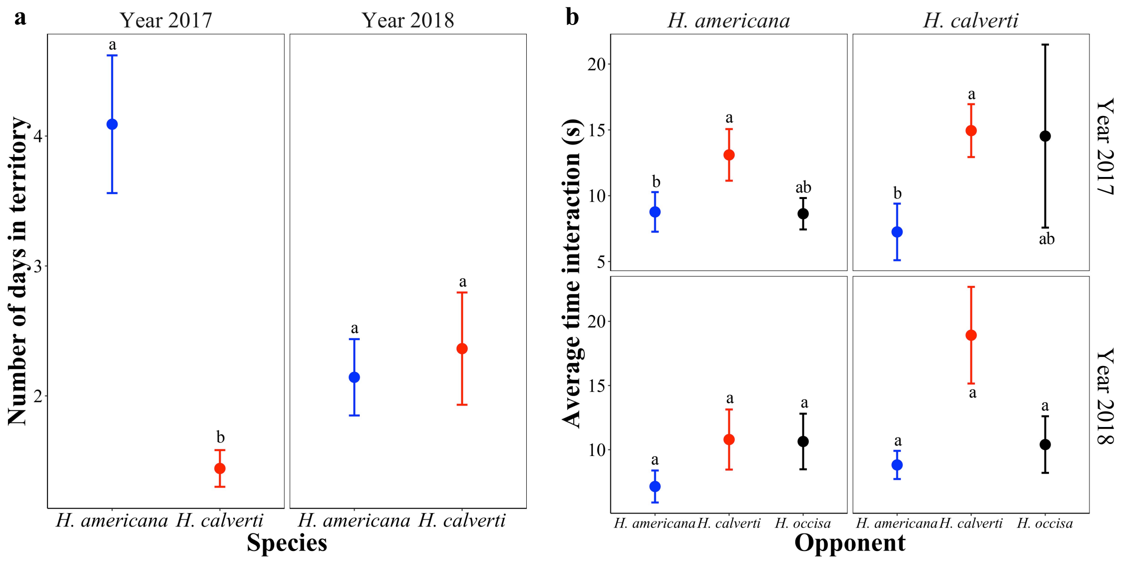

Supplementary Figure S2. Change in male body length between species in allopatry and sympatry. Means and standard errors are shown, different letters represent significant differences.

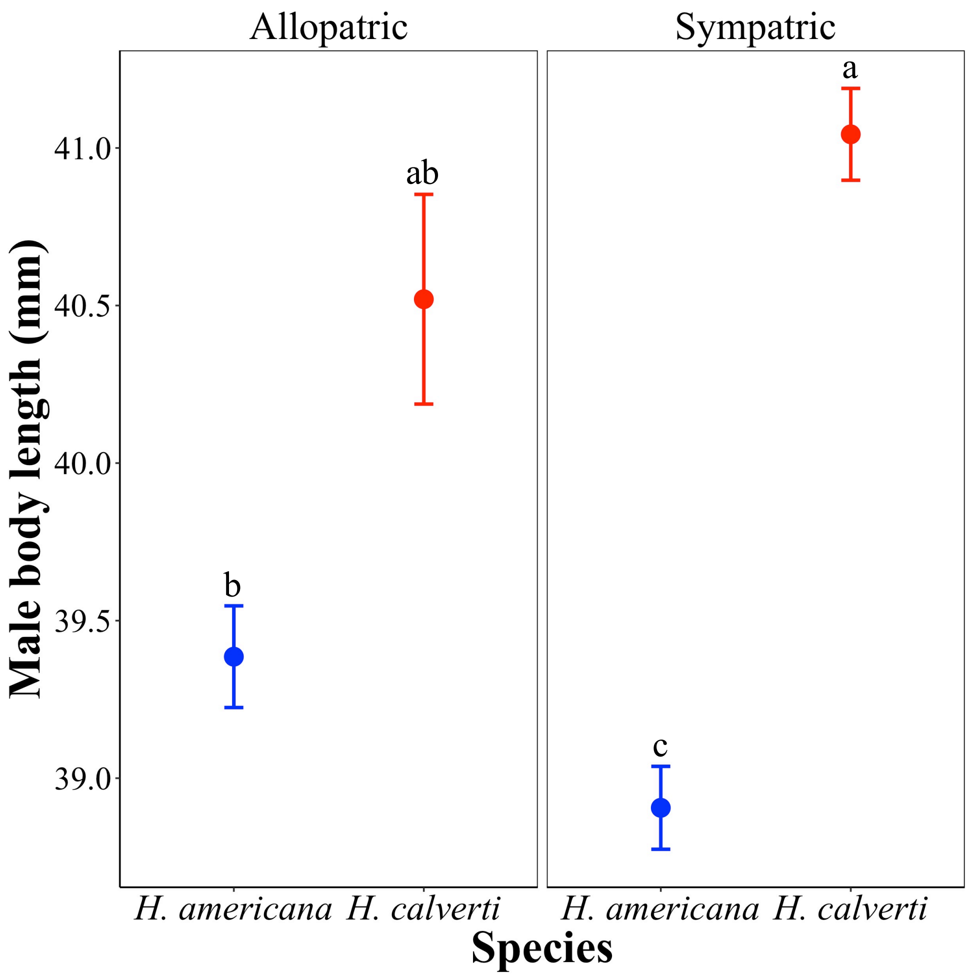

Supplementary Figure S3. Principal component analyses for the wing shape for males and females.

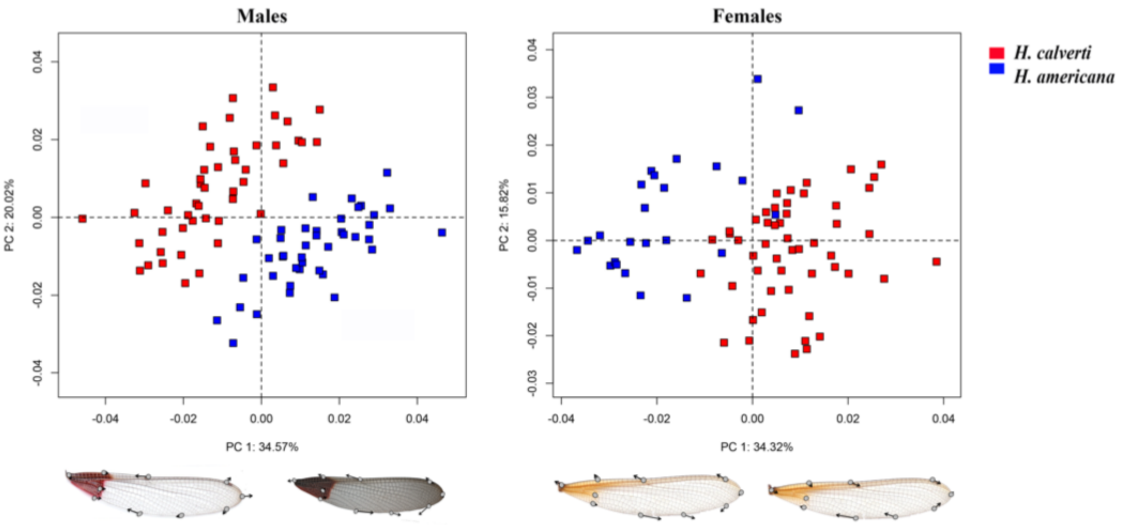

Supplementary Figure S4. Landmarks (green dots) used for geometric morphometric analyses of the wing shape.

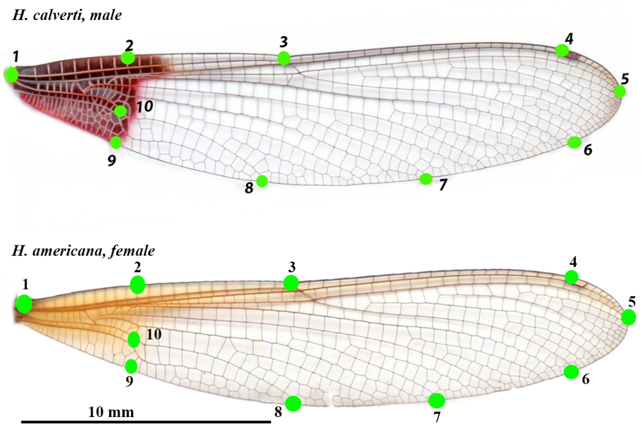

Supplementary Table S1. Non-parametric Wilcoxon analyses for time of interactions between species pair.

Defender	Year	Comparisons	Z	P value
H. americana	2017	*H. americana vs H. calverti*	2.589	0.0096*
	2017	*H. americana vs H. occisa*	1.229	0.2192
	2017	*H. calverti vs H. occisa*	-1.625	0.1042
	2018	*H. americana vs H. calverti*	0.8406	0.4006
	2018	*H. americana vs H. occisa*	1.130	0.2548
	2018	*H. calverti vs H. occisa*	0.4255	0.6704
H. calverti	2017	*H. americana vs H. calverti*	2.8763	0.0040*
	2017	*H. americana vs H. occisa*	1.5712	0.1161
	2017	*H. calverti vs H. occisa*	-0.5315	0.5951
	2018	*H. americana vs H. calverti*	1.9558	0.0505
	2018	*H. americana vs H. occisa*	0.1749	0.8611
	2018	*H. calverti vs H. occisa*	-1.4539	0.1460
*P values < 0.01				

Supplementary Table S2. Summary of wings and body length means and standard errors (SE) for females and males of each species.

Species	Sex	Year	Number of samples	Length (mm)	SE (mm)
Hindwings					
H. americana	Female	2017	10	23.96	0.18
H. americana	Female	2018	7	23.32	0.30
H. americana	Male	2017	20	23.04	0.14
H. americana	Male	2018	17	23.30	0.13
H. calverti	Female	2017	26	25.22	0.19
H. calverti	Female	2018	33	24.63	0.11
H. calverti	Male	2017	17	24.20	0.24
H. calverti	Male	2018	23	23.92	0.20
Forewings					
H. americana	Female	2017	10	25.35	0.18
H. americana	Female	2018	7	24.80	0.32
H. americana	Male	2017	20	24.44	0.15
H. americana	Male	2018	17	24.61	0.16
H. calverti	Female	2017	26	26.75	0.21
H. calverti	Female	2018	33	26.12	0.13
H. calverti	Male	2017	17	25.71	0.24
H. calverti	Male	2018	23	25.33	0.19
Body size					
H. americana	Female	2017	10	34.67	0.28
H. americana	Female	2018	7	32.76	0.41
H. americana	Male	2017	20	38.19	0.31
H. americana	Male	2018	17	37.35	0.24
H. calverti	Female	2017	26	36.73	0.35
H. calverti	Female	2018	33	35.38	0.16
H. calverti	Male	2017	17	41.46	0.51
H. calverti	Male	2018	23	40.42	0.32

Supplementary Table S3. Two-way ANOVA of the differences in the body length, wing length and percentage of wing spots for species, sex and season. d.f.= degrees of freedom; FW=forewing; HW= hindwing.

Variables	d.f.	Sum of squares	F	Prob> F
Body length for females				
Species	1	70.063453	41.5948	<0.0001
Year	1	33.888257	20.1185	<0.0001
Interaction	1	1.005582	0.597	0.4423
FW length for females				
Species	1	23.769169	31.9748	<0.0001
Year	1	4.456791	5.9954	0.0168
Interaction	1	0.018217	0.0245	0.876
HW length for females				
Species	1	21.297432	35.9259	<0.0001
Year	1	4.877674	8.228	0.0054
Interaction	1	0.005624	0.0095	0.9227
Body length for males				
Species	1	184.639	77.9211	<0.0001
Year	1	18.72907	7.904	0.0063
Interaction	1	0.04357	0.0184	0.8925
FW length for males				
Species	1	18.786383	27.0476	<0.0001
Year	1	0.210241	0.3027	0.5839
Interaction	1	1.506546	2.169	0.1451
HW length for males				
Species	1	15.020281	22.6839	<0.0001
Year	1	0.002902	0.0044	0.9474
Interaction	1	1.403016	2.1189	0.1498
Percentage of FW spot				
Species	1	0.00959683	1.794	0.1838
Year	1	0.79971331	149.492	<0.0001
Interaction	1	0.01131587	2.1153	0.1493
Percentage of HW spot				
Species	1	0.01169479	3.5197	0.0639
Year	1	0.60164035	181.0734	<0.0001
Interaction	1	0.00283769	0.854	0.3579

Supplementary Table S4. Procrustes ANOVA results for species, sex and year wing shape variation. d.f. = degrees of freedom; FW=forewing; HW= hindwing; SS= sum if squares; MS= mean square value; Rsq= R-square.

Factor	d.f.	SS	MS	Rsq	F	Z	Prob >F
Male HW							
Year	1	0.003	0.003	0.036	4.25	2.70	0.004
Species	1	0.019	0.019	0.237	27.86	6.96	0.001
Year*species	1	0.0009	0.0009	0.011	1.33	0.71	0.24
Residuals	84	0.059	0.0007	0.715			
Total	87	0.083					
Male FW							
Factor	d.f.	SS	MS	Rsq	F	Z	Pr(>F)
Year	1	0.004	0.003	0.045	5.36	3.39	0.001
Species	1	0.020	0.020	0.227	27.03	6.63	0.001
Year*species	1	0.0009	0.0009	0.010	1.28	0.68	0.265
Residuals	84	0.062	0.0007	0.716			
Total	87	0.087					
Female HW							
Factor	d.f.	SS	MS	Rsq	F	Z	Pr(>F)
Year	1	0.044	0.004	0.073	6.83	3.83	0.001
Species	1	0.008	0.009	0.148	13.78	5.70	0.001
Year*species	1	0.0009	0.001	0.016	1.51	1.04	0.157
Residuals	72	0.046	0.0006	0.762			
Total	75	0.604					
Female FW							
Factor	d.f.	SS	MS	Rsq	F	Z	Pr(>F)
Year	1	0.005	0.006	0.090	8.59	4.25	0.001
Species	1	0.009	0.009	0.144	13.83	5.05	0.001
Year*species	1	0.0006	0.0007	0.011	1.006	0.242	0.406
Residuals	72	0.0487	0.0006	0.754			
Total	75	0.646					

Supplementary Table S5. Summary of body length means and standard errors (SE) for locality and locality type by species. N= Sample size. Data obtained from [14].

Species	Locality	Locality type	N	Mean body length (mm)	SE (mm)
Hetaerina americana	Acahuizotla	Allopatric	26	39.38	0.340
Hetaerina americana	Amacuahutitlan	Allopatric	10	41.23	0.448
Hetaerina americana	Arroando Ajuchitlan	Allopatric	11	38.64	0.401
Hetaerina americana	Arroando Quilamula	Allopatric	32	38.02	0.360
Hetaerina americana	Camichines	Allopatric	9	38.34	0.230
Hetaerina americana	Chupicuaro	Allopatric	10	37.85	0.662
Hetaerina americana	Cocoyotla	Allopatric	7	41.99	0.309
Hetaerina americana	Comitancillo	Allopatric	6	39.89	0.846
Hetaerina americana	El Borbollon	Allopatric	13	37.20	0.538
Hetaerina americana	El Jagueand	Allopatric	7	41.03	0.474
Hetaerina americana	El Limon de Cuauchichinola	Allopatric	5	39.64	0.949
Hetaerina americana	La Mintzita	Allopatric	8	40.28	0.430
Hetaerina americana	La Palma, Huatulco	Allopatric	3	39.42	0.453
Hetaerina americana	La Poza Madre	Allopatric	2	42.94	0.150
Hetaerina americana	Larimer County	Allopatric	3	46.28	0.277
Hetaerina americana	Las Juntas	Allopatric	5	41.37	1.053
Hetaerina americana	Los Yesos	Allopatric	5	41.73	0.738
Hetaerina americana	Momax	Allopatric	7	40.29	0.737
Hetaerina americana	Norte de la posada de la Paz	Allopatric	6	38.02	0.444
Hetaerina americana	Rancho Lo de Campa	Allopatric	10	37.78	0.337
Hetaerina americana	Rio Ayutla, Ejido Santa Maria de los Coocs	Allopatric	2	43.30	1.075
Hetaerina americana	Rio Conchos	Allopatric	7	41.19	0.242
Hetaerina americana	San Agustin	Allopatric	3	35.70	0.734
Hetaerina americana	San Nicolas	Allopatric	8	38.65	0.491
Hetaerina americana	San Pedro Martir	Allopatric	4	36.15	0.926
Hetaerina americana	Tomochic	Allopatric	2	38.83	0.835
Hetaerina americana	Villaldama	Allopatric	8	42.44	0.153
Hetaerina americana	Antiguos Mineros del Norte Las Teclas	Sympatric	2	41.06	2.905
Hetaerina americana	Apazapan	Sympatric	40	37.80	0.199
Hetaerina americana	Chiapa de Corzo	Sympatric	3	38.10	0.456
Hetaerina americana	El Fuerte	Sympatric	2	39.58	0.220
Hetaerina americana	El limon	Sympatric	55	39.38	0.315
Hetaerina americana	Ixtlahuacan	Sympatric	9	39.87	0.516
Hetaerina americana	Jiliotupa	Sympatric	4	40.92	1.058
Hetaerina americana	Nuevo Recuerdo	Sympatric	6	39.90	0.451
Hetaerina americana	Puente Ayutla	Sympatric	2	40.92	0.135
Hetaerina americana	Puente, Arroando Guajiniquil	Sympatric	5	39.90	0.738
Hetaerina americana	San Pedrito Chicozapote	Sympatric	11	39.19	0.667
Hetaerina americana	Santiago Dominguillo	Sympatric	72	38.63	0.183
Hetaerina calverti	Arroyo, Poza Azul	Allopatric	4	37.17	0.868
Hetaerina calverti	Cascada de Micos al NO de Cd. Valles	Allopatric	4	42.18	0.560
Hetaerina calverti	Churince Poza Churince	Allopatric	5	39.55	0.549
Hetaerina calverti	El Platanal	Allopatric	7	40.74	0.357
Hetaerina calverti	La Poza Azul	Allopatric	3	40.12	0.471
Hetaerina calverti	Pisaflores	Allopatric	2	41.76	0.390

Hetaerina calverti	Rancho Orozco Poza Escobedo	Allopatric	2	41.29	2.800
Hetaerina calverti	Rio Negro, Sacapulas	Allopatric	5	40.82	0.410
Hetaerina calverti	Rio San Marcos	Allopatric	3	42.43	1.272
Hetaerina calverti	Antiguos Mineros	Sympatric	7	40.32	0.827
Hetaerina calverti	Apazapan	Sympatric	42	40.84	0.278
Hetaerina calverti	Chiapa de Corzo	Sympatric	2	40.79	0.435
Hetaerina calverti	El limon	Sympatric	20	41.90	0.444
Hetaerina calverti	Nuevo Recuerdo	Sympatric	3	42.74	0.739
Hetaerina calverti	Puente Ayutla	Sympatric	7	41.83	0.554
Hetaerina calverti	Arroando Guajiniquil	Sympatric	7	40.42	0.363
Hetaerina calverti	San Pedrito Chicozapote	Sympatric	2	39.64	0.545
Hetaerina calverti	Santiago Dominguillo	Sympatric	41	40.80	0.234
Hetaerina calverti	Zaragoza	Sympatric	6	41.63	0.282
